# Serum biomarkers and traditional risk factors as predictors of peripheral arterial disease assessed by magnetic resonance angiography

**DOI:** 10.1186/1532-429X-11-S1-P124

**Published:** 2009-01-28

**Authors:** Yuliya B Goldsmith, Ijaz Ahmad, Vishnu Singh, Marcus D'Ayala, Igor Klem, William M Briggs, Mohammed Ahmed, Munawar Hayat, Terrence J Sacchi, John F Heitner

**Affiliations:** 1 New York Methodist Hospital, Brooklyn, NY USA; 2 Duke University Medical Center, Durham, NC USA

**Keywords:** Magnetic Resonance Angiography, Peripheral Arterial Disease, Left Ventricular Dysfunction, Traditional Risk Factor, Multivariate Regression Model

## Introduction

In recent studies, multiple serum biomarkers had been investigated for a relationship to peripheral arterial disease (PAD). PAD presence and severity have been shown to be associated with CRP and lipid levels. Although elevated pro-BNP has clearly been shown to be associated with left ventricular dysfunction and predict cardiovascular events, there is scant data showing its association with PAD. Prior small studies demonstrated elevated levels of NT-pro BNP in patients with PAD when compared to controls. The significance of the serum biomarkers as predictors of PAD in comparison with traditional risk factors is unclear.

## Purpose

The aim of this study is to compare the role of serum pro-BNP, CRP, and lipid levels to the number of standard risk factors (nRF) as independent predictors of the presence and severity of PAD.

## Methods

We prospectively evaluated 205 consecutive patients who underwent contrast-enhanced MRA for the evaluation of PAD. There were 71 carotid (10 segments), 131 abdominal (2 segments), 115 renal (2 segment), 66 pelvic (6 segments), and 48 lower extremity studies (12 segments). We analyzed the studies for both the presence and the severity of PAD. Significant PAD was defined as any stenosis of >50% in at least one vessel. PAD severity was assessed with fractional stenosis score (FSS): patients segment scores (0 = no stenosis, 1 = 1–50%, 2 = 51–75%, 3 = 76–99%, 4 = 100%) were summed and divided by the maximal possible sum score that would have resulted if all evaluated segments were occluded. nRF for each patient was calculated by assigning 1 point for the presence of each: age >65, male gender, BMI >25, hypertension, diabetes, hyperlipidemia and smoking. Serum pro BNP, CRP and lipid profile values were collected on the day of the CMR for each patient.

## Results

There were 124 pts with significant PAD. In a univariate model, serum log (pro BNP), total cholesterol (TC), LDL, triglycerides (TG), and nRF were associated with FSS. Log (pro BNP), TC, LDL, and nRF were associated with presence of significant PAD. CRP was not predictive of either presence or severity of PAD. When a multivariate regression model was applied, only log (pro BNP) (p < 0.027), nRF (p < 0.007), and LDL (P < 0.004) were independently associated with FSS. When accounting for the presence of systolic and distolic dysfunction by echocardiogram (99 pts), log (pro BNP) remained a significant predictor of FSS. Only nRF was independently associated with the presence of significant PAD (OR = 1.09, 95% CI 1.04–1.15, p < 0.0008 for each additional RF).

## Conclusion

Although log (pro BNP) and LDL were associated with severity of PAD expressed as FSS, only nRF was independently predictive of both presence and severity of PAD. The role of serum pro BNP as a marker of PAD needs to be further investigated. In this study, serologic biomarkers do not add to traditional risk factors for the prediction of PAD diagnosed with MRA.

